# Targeting Melanogenesis with Postbiotics: An Integrated Zebrafish-Based Assessment of *Lactobacillus salivarius* BGHO-1 and *Lactobacillus paracasei* BGSJ2-8

**DOI:** 10.3390/molecules30204134

**Published:** 2025-10-20

**Authors:** Gabor Katona, Natasa Jovanovic Ljeskovic, Ivana Strahinic, Nemanja Stanisavljevic, Sladjana Vojvodic, Jelena Djuris, Aleksandar Pavic

**Affiliations:** 1 Faculty of Pharmacy Novi Sad, University Business Academy in Novi Sad, Heroja Pinkija 4, 21101 Novi Sad, Serbia; gabor.katona@ffns.ac.rs (G.K.); natasa.ljeskovic@ffns.ac.rs (N.J.L.); sladjana.vojvodic@ffns.ac.rs (S.V.); 2 Institute of Molecule Genetics and Genetic Engineering, University of Belgrade, Vojvode Stepe 444a, 11042 Belgrade, Serbia; ivana.stahinic@imgge.bg.ac.rs (I.S.); nemanja.stanisavljevic@imgge.bg.ac.rs (N.S.); 3 Faculty of Pharmacy, University of Belgrade, Vojvode Stepe 450, 11221 Belgrade, Serbia; jelena.djuris@pharmacy.bg.ac.rs

**Keywords:** *Lactobaccilus salivarius*, *Lactobacillus paracasei*, melanogenesis, zebrafish, melanocytes imaging

## Abstract

Skin hyperpigmentation disorders represent a major dermatological challenge, and safe alternatives to conventional depigmenting agents remain scarce. Probiotics and their postbiotic derivatives have emerged as promising natural candidates; however, only a few bacterial strains have been investigated for melanogenesis-inhibitory activity, and their true potential remains largely unexplored. Here, we report for the first time the biosafety profile and anti-melanogenic activity of *Lactobacillus salivarius* BGHO-1 and *Lactobacillus paracasei* BGSJ2-8, and assess their possible use in the treatment of skin hyperpigmentation. Two complementary zebrafish-based approaches were employed: (i) image-assisted analysis of pigmentation patterns, melanocyte morphology, and melanocytotoxicity, and (ii) quantitative melanin analysis, enabling integrated safety and efficacy evaluation. We investigated both native and heat-inactivated preparations, including whole cultures, cell-free supernatants, isolated cells, and separated cell walls/membranes and cytoplasmic fractions. While several fractions demonstrated the ability to inhibit melanogenesis, the cell wall/membrane fraction was the most potent, reducing melanin content by 64% compared to untreated embryos, while causing no systemic side effects and preserving melanocyte structure. Furthermore, this fraction did not elicit inflammatory responses or neutropenia, underscoring its favorable safety profile at anti-melanogenic doses. Collectively, this study identifies specific postbiotics as effective and safe modulators of melanogenesis and highlights their translational potential in developing novel approaches for treating skin hyperpigmentation.

## 1. Introduction

Melanin production—melanogenesis—is a tightly regulated physiological process in which specialized skin cells, melanocytes, synthesize the pigment melanin via the activity of the multicopper enzyme tyrosinase [1]. This pigment plays a central role in determining skin and hair color in mammals, including humans [2]. The regulation of skin pigmentation involves a complex interplay of molecular pathways, with the key processes responsible for melanin synthesis and its control, melanosome transfer, and the activation of melanocytes. Skin pigmentation is governed by a complex and coordinated interplay of molecular pathways such as MC1R signaling, KIT-SCF, Wnt/β-catenin, endothelin signaling, MAPK/ERK, and PKC pathway, which upregulate genes encoding major enzymes involved in melanin synthesis: tyrosinase (TYR), tyrosinase-related protein 1 and 2 (TYRP1, TYRP2), and dopachrome tautomerase (DCT) [3,4]. Moreover, this biological process is tightly regulated by the neuro-immuno-endocrine system, involving several neural signals, neurotransmitters, and neuropeptides (e.g., melanocyte-stimulating hormone-α-MSH, β-endorphin, adrenocorticotropic hormone-ACTH), together with hormones (e.g., melatonin), as well as pro-inflammatory cytokines (IL-18, IL-33, IFN-γ) and prostaglandins, which significantly modulate the melanin biosynthesis in the skin [5,6,7].

Beyond pigmentation, melanin serves critical protective functions, most notably by shielding the skin from harmful ultraviolet (UV) radiation and reducing the risk of skin carcinogenesis [8]. However, dysregulation of melanogenesis, particularly the excessive accumulation of melanin, can lead to various pigmentary disorders such as freckles, melasma, ephelides, senile lentigines, melanoderma, and chloasma, which is a burden that is emerging at the global level.

Melasma shows marked geographic and ethnic heterogeneity, with reported prevalence reaching ~40% in high-risk populations (i.e., some Southeast Asian cohorts), implying tens of millions of affected individuals in the most affected regions [9]. Post-inflammatory hyperpigmentation (PIH) is also highly prevalent, particularly among persons with darker phototypes [10], and constitutes a frequent complication of acne, eczema, and cutaneous procedures in these populations [11]. On the other side, solar lentigines (“age spots”) become increasingly prevalent in older adults, affecting more than 90% of individuals older than 50 years [12]. The burden of pigmentary disorders is additionally amplified by chronic ultraviolet (UV) exposure and biological aging, mediating cumulative DNA damage, oxidative stress, and altered melanocyte behavior that promote focal hyperpigmentation [13].

While many forms of skin hyperpigmentation are primarily aesthetic in nature, they can significantly affect psychological well-being. The visible nature of these lesions often impacts self-esteem and emotional health, ultimately impairing overall quality of life [14,15,16]. In addition, hyperpigmentation is frequently accompanied by inflammatory responses such as eczema and contact dermatitis and may even be associated with increased skin cancer susceptibility [14,17]. Given the clinical and psychological implications of skin hyperpigmentation, the identification of new agents capable of safely and effectively suppressing melanin synthesis represents a crucial step toward the development of next-generation treatments for pigmentation disorders.

A wide spectrum of melanogenesis inhibitors has been identified to date, acting through distinct molecular mechanisms. Compounds like kojic acid, hydroquinone, and arbutin exert their effects by directly inhibiting the enzymatic activity of tyrosinase, which has a critical role in melanin synthesis and was recognized as a key target for the screening and discovery of novel anti-melanogenic bioactive agents [18]. Others downregulate tyrosinase gene expression, as observed with resveratrol and quercetin [19,20], or interfere with melanosome maturation and transport, exemplified by niacinamide [21]. In addition, antioxidants such as vitamin C can neutralize reactive oxygen species that promote UV-driven signaling of melanogenesis [22]. Other pigmentation-reducing agents can act by causing the exfoliation of pigmented cells, such as fruit acids (glycolic, salicylic, lactic acid, etc.) and retinoids (retinol, retinal, tretinoin, adapalene, etc.) [23,24]. However, despite the mechanistic diversity of these agents, many melanogenesis-inhibiting agents are hindered for long-term use due to their inherent drawbacks, such as poor chemical stability (kojic acid and ascorbic acid), irreversible cytotoxic effects leading to melanocyte loss (hydroquinone) [25], and insufficient depigmenting efficacy (niacinamide) [21]. Moreover, some of the most widely used compounds, particularly hydroquinone and kojic acid, have been shown to possess mutagenic, clastogenic, and even carcinogenic potential [26], raising significant safety concerns. Moreover, the FDA banned the use of hydroquinone in cosmetic products in 2020. These limitations highlight the urgent and pressing demand in the dermatology practice and cosmetic industry for the development of novel melanogenesis inhibitors that offer an optimal balance between efficacy, safety, and stability for both therapeutic and cosmetic applications.

Recent studies focusing on microorganism-derived agents have identified specific probiotic bacteria and their derivatives as promising alternatives for the effective and safe treatment of skin hyperpigmentation disorders [27,28,29,30,31,32,33,34,35,36,37,38,39] (Table 1). The human skin microbiome is currently the subject of intensive research aimed at elucidating its role in the pathogenesis of various skin conditions, including hyperpigmentation [40]. Although the effects of exogenously administered probiotics, whether oral or topical, are not yet fully understood due to inter-individual variability in skin microbiota composition, there is increasing evidence supporting their positive impact on skin health [41]. A growing number of skincare products on the market now incorporate probiotics, predominantly lactic acid bacteria, as well as their bioactive derivatives such as prebiotics, postbiotics, and parabiotics. These compounds have shown considerable promise in terms of both safety and efficacy, making them attractive candidates for skin health applications [42]. Reported benefits include improvements in conditions associated with microbial dysbiosis and immune dysregulation, such as acne, eczema, and psoriasis, as well as cosmetic effects like anti-aging, antioxidative, and antimicrobial activities [43].

Probiotic and postbiotic metabolites can influence melanogenesis through multiple mechanisms, including antioxidant activity that reduces oxidative stress in the epidermal niche, modulation of local immune and inflammatory signaling that secondarily affects melanocyte activation, and direct suppression of melanogenic mediators such as MITF and tyrosinase. Consistent with these mechanisms, recent in vitro studies report that cell-free supernatants or defined postbiotic components from lactic acid bacteria reduce α–MSH–induced melanin synthesis, inhibit tyrosinase activity, and downregulate expression of melanogenesis-related genes such as MITF, TYR, TRP1/2, supporting their candidacy as safe depigmenting agents for further translational evaluation [28,29,39].

Given the limitations of conventional anti-melanogenic agents and the growing body of evidence demonstrating that probiotic-derived compounds may modulate skin pigmentation, this study aimed to evaluate the anti-melanogenic activity, biosafety, and immunotoxicity of postbiotic fractions from *Lactobacillus salivarius* BGHO-1 and *Lactobacillus paracasei* BGSJ2-8 using zebrafish as an in vivo platform. To this end, we employed the two complementary zebrafish-based approaches to assess their anti-melanogenic activity alongside melanocytotoxicity and immunotoxicity responses, as well as to perform imaging-based analyses of melanocytes’ morphological characteristics. In particular, we leveraged the zebrafish (*Danio rerio*) model as a powerful and transparent preclinical platform allowing for high-throughput evaluation of both the safety and efficacy of the tested probiotics and their postbiotic derivatives. In contrast to conventional assays that utilize mushroom tyrosinase, an enzyme structurally distinct from its human counterpart and thus poorly predictive of outcomes in human skin [25], the zebrafish model enables physiologically relevant whole-organism insights into melanogenesis inhibition.

## 2. Results and Discussion

Considering that the current dermatological and cosmetics practice face a significant gap in achieving effective but safe treatment of pigmentation-related skin disorders [25,44] and that probiotics and their derivatives (prebiotics, postbiotics, and parabiotics) have attracted a huge attention as safe biological alternative to chemical anti-melanogenic and depigmenting agents, we first conducted a systematic literature search to comprehensively survey probiotic bacterial strains with inhibitory effects on melanin synthesis. We focused on studies examining the effects of viable bacterial cells (probiotics), inactivated cells (parabiotics), and their cell wall fragments, metabolic by-products, and extracellular metabolites (postbiotics) (Table 1), while excluding studies that investigated fermentation products enriched with probiotics. To the best of our knowledge, only 15 probiotic species have been investigated to date, belonging to the genera *Lactobacillus* (10), *Bifidobacterium* (4), and *Pediococcus* (1) (Table 1). In most of these studies, anti-melanogenic activity was assessed almost exclusively in cell-free supernatants derived from native bacterial cultures, with only limited attention given to heat-inactivated cells and their constituents (postbiotics) (Table 1). Moreover, the majority of investigations relied on oversimplified in vitro models, such as murine B16 melanoma cells or mushroom tyrosinase assays, which lack physiological relevance and do not adequately reflect the complexity of pigmentation biology [25].

Seeking to identify novel probiotic-based agents with potent anti-melanogenic activity, we carried out a comprehensive investigation using the two well-characterized probiotic strains—*L. salivarius* BGHO-1, isolated from the human oral cavity [45], and *L. paracasei* BGSJ2-8, isolated from a traditional homemade semi-hard cheese product [46]. Both strains have previously been demonstrated to exert a broad spectrum of health-promoting effects, including antimicrobial activity, immune stimulation, anti-inflammatory and antioxidant properties, as well as the ability to improve mitochondrial function [47,48,49]. To the best of our knowledge, however, neither of these two species has been comprehensively examined to date for anti-melanogenic potential or possible application in managing skin hyperpigmentation disorders.

**Figure 1:**
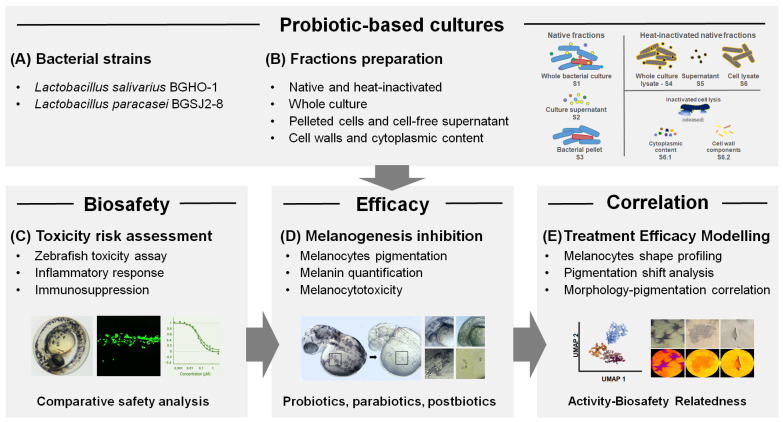
Uncovering the melanogenesis-inhibitory potential of two probiotic strains, *L. salivarius* BGHO-1 and *L. paracasei* BGSJ2-8. Schematic overview of the experimental pipeline based on functionalized zebrafish assays. (**A**) Selected bacterial strains from the laboratory collection were cultivated in MRS broth, and (**B**) processed into the eight fractions, comprising both native (untreated) fractions (S1–S3) and heat-inactivated fractions (S4–S6.2) that included cellular and extracellular components. (**C**) The biosafety profile of each fraction was assessed in vivo using the zebrafish model, through toxicity assays, evaluation of inflammatory responses, and immunosuppression readouts. In parallel, their melanogenesis-inhibitory potential (**D**) was evaluated through complementary zebrafish-based approaches: image-assisted analysis of melanocytes and quantitative analysis of extracted melanin. Finally, (**E**) treatment efficacy was modeled by integrating safety and activity data, correlating morphological changes with pigmentation outcomes to determine activity-biosafety relatedness.

In this study, a fractionation of bacterial material has been applied, encompassing live and heat-inactivated bacterial cultures, pelleted bacterial cells, cell-free supernatants, and distinct cellular fractions including cell wall/membrane components and membrane-free cytoplasmic content. Importantly, beyond this fraction-level dissection, we evaluated all treatments in vivo using the zebrafish (*Danio rerio*) model, which provides a physiologically relevant platform for the simultaneous assessment of the selected probiotic strains and their impact on melanin biosynthesis, melanocyte integrity, toxicity, and immune response. This integrated strategy enabled a more refined characterization of probiotic-derived fractions with anti-melanogenic activity and the identification of specific subcellular contributors underlying the observed effects (Figure 1). As far as we know, this is the first study to systematically examine both the melanogenesis-inhibitory effects and biocompatibility of probiotic strains and their derivatives using the zebrafish model, marking a significant step toward advancing methodologies in this area.

The zebrafish model represents a versatile and high-throughput preclinical platform, making it highly suitable for the discovery of effective and safe bioactive compounds, including inhibitors of melanogenesis. Owing to its genetic, physiological, and immunological similarities to humans, zebrafish has emerged as a reliable alternative to conventional mammalian models, facilitating translational research and reducing the risk of late-stage failures [50]. The optical transparency of zebrafish embryos enables simultaneous, real-time, multiparametric evaluation of systemic toxicity and direct visualization of treatment effects on skin melanocyte pigmentation and integrity [51]. In addition, the increasing regulatory restrictions on animal testing for cosmetic products, particularly within the European Union, underscore the importance of zebrafish embryos as an ethical and scientifically robust alternative. By integrating safety and efficacy evaluation in real time, the zebrafish model is being increasingly incorporated into preclinical screening pipelines for depigmenting agents and cosmetic formulations, thus providing a powerful bridge between mechanistic research and clinical translation.

As a first step, zebrafish embryos were exposed to the different doses of both native (untreated) fractions (S1–S3) and heat-inactivated fractions (S3–S6) of BGHO-1 and BGSJ2-8, including whole cultures (S1 and S4, respectively), cell-free supernatants (S2 and S5, respectively), and pelleted bacterial cells (S3 and S6, respectively) (Figure 1). Embryos were treated at the 6-hpf developmental stage, ensuring high sensitivity to the tested agents, and carefully inspected the survival, development, and inner organs functioning (Table S1) over a period of 5 consecutive days (up to 120 hpf). Based on these data, we determined the major toxicological parameters, LC_50_ and EC_50_, which indicate the concentrations causing mortality or adverse effects in 50% of exposed embryos. Particular attention has been paid to the pigmentation phenotype of the treated embryos, as well as the appearance of signs of melanocytotoxicity.

**Figure 2:**
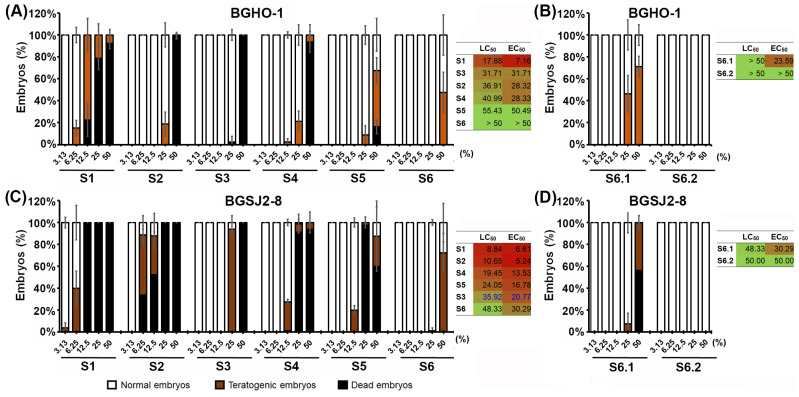
Toxicological profile of the different fractions of the two strains *L. salivarius* BGHO-1 (**A**,**B**) and *L. paracasei* BGSJ2-8 (**C**,**D**) studied in vivo using the zebrafish embryo model, expressed as the dose-dependent effect on survival and occurrence of adverse effects after 5-day treatment. The effect of native (S1–S3) and heat-inactivated (S4–S6.2) fractions was tested, including whole bacterial cultures (S1 and S4), their supernatants (S2 and S5), pelleted bacterial cells (S3 and S6), the membrane-free cytoplasmic fraction (S6.1), and the fraction containing cell wall/membranes (S6.2). Relevant toxicological parameters—LC_50_ and EC_50_ values are determined for each fraction tested.

We found that the heat-inactivated fractions (S4–S6) of both strains exhibited a much favorable toxicological profile than their native counterparts (S1–S3), with the treatments from BGHO-1 causing fewer adverse effects than those from BGSJ2-8 (Figure 2A,B). For both bacterial strains, out of the six fractions tested (S1–S6), the S6 fractions showed the best safety profile, with no adverse side effects at doses up to 50%. On the other side, the fractions S1 and S2—corresponding to the native cultures and the given supernatants, respectively—induced the worst toxicity response (Figure 2), mostly manifested as body growth retardation and tail necrosis (Figures S1 and S2).

Given that lactobacilli are known to secrete bacteriocins and other biologically active proteinaceous molecules at very low micromolar concentrations [52], the observed toxicity of native supernatants was ultimately not surprising. In contrast, heat-inactivation of the given supernatants (90 °C) most likely resulted in protein denaturation and loss of extracellular bioactivity, thereby contributing to the improved safety profile of the heat-treated formulations. These data underscore the importance of distinguishing between native preparations and their fractionated counterparts, as the separation of cellular and secreted components is crucial to disentangle true anti-melanogenic effects from non-specific cytotoxic responses.

Since the S6 fraction exhibited the most favorable in vivo safety profile for both tested strains, we further segregated the inactivated bacterial cells into two components: a membrane-free cytoplasmic fraction (S6.1) and a fraction containing only cell walls and membranes (S6.2). Data obtained after a 5-day treatment showed that the S6.2 fraction, from both strains, induced no detectable side effects in zebrafish embryos even at the highest applied concentration of 50% (LC_50_ > 50%, EC_50_ > 50%), while the S6.1 fractions induced a weak toxic response (Figure 1B,D). Importantly, the S6.1 fraction still displayed a considerably better safety profile compared with the S1-S6 fractions, as reflected by substantially higher LC_50_ and EC_50_ values.

### 2.1. L. salivarius BGHO-1 and L. paracasei BGSJ2-8 Possess Melanogenesis-Inhibiting Activity

As the developing zebrafish embryo possesses clearly discernible melanocytes within the skin epidermis, we evaluated, in parallel with the toxicity assessment, the effect of fractions S1–S6.2 on melanin synthesis and melanocyte morphology. Kojic acid and hydroquinone, two well-known inhibitors of melanogenesis used in the treatment of hyperpigmentation disorders [53], were used as reference controls.

**Figure 3:**
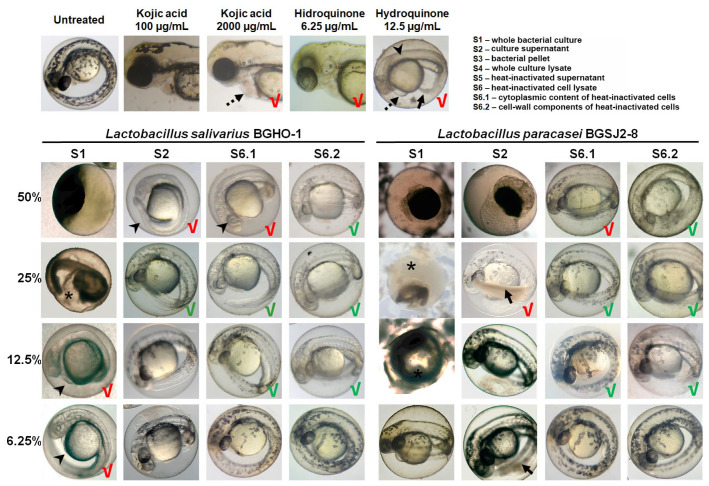
The dose-dependent inhibition of melanogenesis in zebrafish embryos exposed to the selected fractions derived from bacterial cultures of *L. salivarius* BGHO-1 and *L. paracasei* BGSJ2-8. Representative images of treated embryos (*n* = 50 per treatment, three independent experiments) are shown for the indicated fractions (S1, S2, S6.1, and S6.2). Non-toxic treatments that reduced embryo pigmentation without adverse effects are marked with a green tick on the corresponding images. The treatments that provoked toxic side effects are marked with a red tick, including embryo mortality (asterisk), impaired growth and development (arrowhead), tail necrosis (solid arrow), pericardial edema (dashed black arrow).

We found that both BGHO-1 (Figure S1) and BGSJ2-8 (Figure S2) robustly inhibit melanogenesis in zebrafish embryos. This effect was observed across multiple fractions derived from overnight cultures (S1), although the magnitude of reduced pigmentation correlated closely with each fraction’s safety profile. Fractions S2, S4, S5, S6, S6.1, and S6.2 from both strains significantly reduced the embryonic skin pigmentation relative to untreated controls; however, several of these fractions—most notably S2, S4, and S5—also impaired embryonic development and induced tail necrosis at anti-melanogenic doses, limiting their translational potential. Figure 3 shows representative images for a selected subset of treatments (S1, S2, S6.1, and S6.2) illustrating these contrasting safety-efficacy profiles. We found that the S6.2 fraction consistently produced the strongest inhibition of melanocyte pigmentation while preserving normal embryonic development. Embryos exposed to the concentrations of 12.5–25% of S6.2 were largely depigmented, particularly following BGHO-1 treatment (Figure 3), demonstrating an efficient inhibition of melanin synthesis. The related fraction S6.1 also reduced pigmentation but caused mild toxicity at higher concentrations (50%).

Similarly, the treatments with kojic acid and hydroquinone also reduced pigmentation in zebrafish embryos. However, kojic acid required relatively high concentrations (≥2000 µg/mL) to produce substantial inhibition of skin melanogenesis, at which point cardiotoxic effects were observed (Figure 3), consistent with our previous work [54]. Hydroquinone, in contrast, showed strong anti-melanogenic activity at low concentrations (6.25 µg/mL) but was associated with pronounced developmental toxicity, particularly visible at doses that completely abolished pigmentation (12.5 µg/mL) (Figure 3).

Together, these results indicate that multiple bacterial components contribute to the anti-melanogenic activity of BGHO-1 and BGSJ2-8, and that efficacy is not only restricted to whole probiotic cells or extracellular metabolites. Importantly, the postbiotic fraction enriched in membrane fragments from heat-inactivated probiotic cells possesses both potent depigmenting activity and a favorable in vivo safety profile. These findings position membrane-enriched postbiotic fractions as promising next-generation candidates for further development and justify the isolation of active principle(s), targeted molecular characterization, mechanism-of-action studies, and validation in mammalian and human-relevant models.

**Figure 4:**
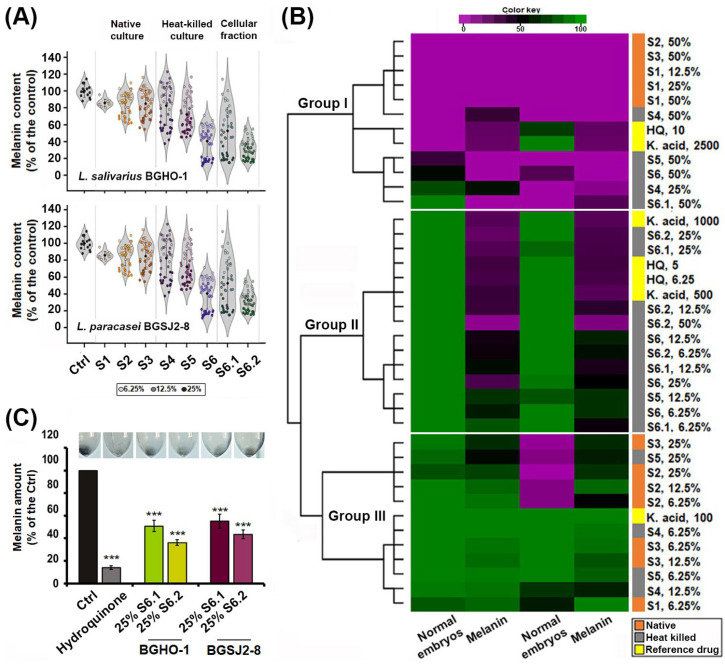
Assessment of the anti-melanogenic potential of *L. salivarius* BGHO-1 and *L. paracasei* BGSJ2-8 fractions using imaging-based and melanin extraction approaches. (**A**) Melanin content in embryonic melanocytes was quantified using RGB-based thresholding techniques (*n* = 6 embryos per group, with 3–5 melanocytes analyzed per embryo). Dot color shade/intensity indicates-darkest (25%), lighter (12.5%), and lightest (6.25%). (**B**) Cluster analysis of the treatments, based on melanin content and in vivo toxicity data, clearly distinguished safe and effective treatments (Group II) from effective but unsafe treatments (Group I) and safe but ineffective treatments (Group III). (**C**) Depigmenting activity of fractions S6.1 and S6.2 from both strains was confirmed by melanin extraction from treated embryos (50 pooled embryos per group). The amount of melanin after applied treatments is shown in relation to the untreated control group and expressed as the mean ± SD of the independent experiments. Kojic acid and hydroquinone were included as reference anti-melanogenic agents. Statistically significant differences in melanin content between control and treated groups are indicated (*** *p* < 0.001, ANOVA and Bonferroni test). Representative images display the melanin content in pelleted embryonic melanocytes after each treatment.

To further compare the anti-melanogenic efficacy of probiotic-derived treatments, we employed two complementary zebrafish-based approaches: (i) image-assisted melanogenesis assessment and (ii) quantification of extracted melanin. While the extraction and quantification of total melanin from pooled zebrafish embryos (50–100 per group) provides a reliable measure of overall pigmentation differences between treatment groups, this bulk assay did not reveal spatial patterns of depigmentation or melanocyte-specific morphological changes, and scaling it to compare all fractions (S1–S6) would require several thousand embryos. To overcome these limitations, we used a complementary imaging-based strategy to simultaneously assess the following: (i) relative melanin content, (ii) melanocyte size and morphology, (iii) spatial distribution of pigmentation, and (iv) potential melanocytotoxicity. By integrating these two levels of analysis, we gained a more comprehensive insight into treatment effects, enabling us to distinguish true anti-melanogenic activity from hypopigmentation caused by melanocytotoxicity and thus identify treatments that reduce pigmentation while preserving melanocyte structure and function (Figure 4).

We first imaged zebrafish embryos by light microscopy and quantitatively analyzed pigmentation levels in individual melanocytes following eight different treatments applied at three doses (6.25%, 12.5%, and 25%), using RGB-based thresholding techniques implemented in ImageJ v1.54a. Image analysis, illustrated by violin plots of individual melanocyte pigmentation (Figure 4A), confirmed the strongest depigmenting effects for S6.2 and S6.1. We then performed a hierarchical cluster analysis by integrating melanin content data obtained from image-based quantification with in vivo toxicity profiles generated in the previous assay (Figure 4B). This combined approach enabled comparison of probiotic-derived treatments not only among themselves but also against the benchmark anti-melanogenic compounds kojic acid and hydroquinone. The performed clustering segregated all treatments into three distinct groups (Figure 4B): (i) toxic treatments with strong inhibitory effects on skin pigmentation (including native S1, S2 and S3 fractions at high doses, as well as kojic acid and hydroquinone), (ii) safe or minimally toxic treatments with clear anti-melanogenic activity (including fractions S6, S6.1 and S6.2), and (iii) ineffective and non-toxic treatments, with no measurable effect on melanin synthesis (native and heat-inactivated S1–S5 fractions at doses < 25%).

After confirming that fractions S6.2 and S6.1 reduced melanin synthesis while preserving melanocyte integrity, we extracted and quantified melanin from pooled samples of 50 embryos per treatment (Figure 4C). The analysis showed that the melanin content was reduced by approximately 58% with the S6.1 fraction of the BGSJ-8 culture and by slightly more (64%) with the corresponding fraction derived from the BGHO-1 culture, compared with untreated controls. These findings are in line with the reductions in melanin levels previously observed using the imaging-based strategy.

### 2.2. L. salivarius BGHO-1 and L. paracasei BGSJ2-8 Do Not Provoke Melanocytotoxic Response In Vivo

As several postbiotic fractions from both probiotic strains reduced pigmentation in zebrafish embryos, we next asked whether this effect is due to true inhibition of melanogenesis or rather reflects a negative impact on melanocyte development and function (melanocytotoxicity). To address this question, we analyzed the morphological features of individual melanocytes in exposed embryos using the same RGB-based thresholding techniques, with the aim of assessing both their structural integrity and spatial melanin distribution. Under physiological conditions, melanocytes display dendritic, star-shaped projections that are essential for transferring melanin to surrounding keratinocytes. Loss of dendricity and cell rounding are widely recognized as hallmarks of melanocytotoxicity [55]. To capture such structural changes, we measured two key morphometric descriptors: solidity and the area-to-perimeter ratio. Solidity, ranging from 0 to 1, reflects the degree of melanocyte branching, with lower values indicating a more complex dendritic morphology. The area-to-perimeter ratio captures overall cell shape and compactness, where lower values indicate highly branched melanocytes, while higher values reflect reduced dendricity and compromised morphology, which are critical for melanin transfer.

**Figure 5:**
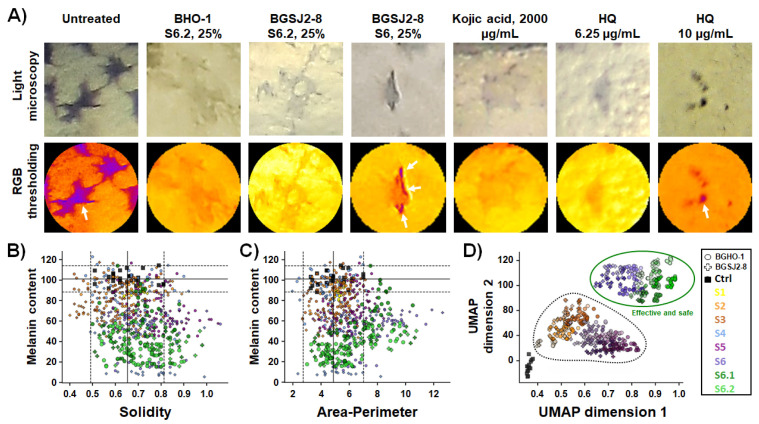
Evaluation of melanocytotoxic activity of *L. salivarius* BGHO-1 and *L. paracasei* BGSJ2-8 fractions by imaging-based morphometric analysis. (**A**) Representative images showing melanocyte morphology and melanin distribution in zebrafish embryos after 48 h of treatment with different fractions. Non-toxic treatments have no impact on melanocyte morphology assessed by solidity and area-perimeter parameters (25% S6.2 of BGHO-1 and BGSJ2-8; 2000 µg/mL kojic acid), whereas toxic treatments caused loss of dendricity (25% S6 of BGSJ2-8; 6.25 µg/mL hydroquinone—HQ) or cell rounding (10 µg/mL HQ). Arrows highlight peripheral melanin accumulation at the membrane boundaries of melanocytes that have lost dendricity. (**B**,**C**) Effects of treatments on melanin content normalized to morphometric descriptors (solidity and area-to-perimeter ratio). Each data point corresponds to one melanocyte measurement. Data points are color-coded by the applied treatments: probiotic strains, BGHO-1 = circles, BGSJ2-8 = crosses. Dashed lines represent ± 2 SD from control means, serving as thresholds for normal morphological characteristics and melanin level found in the control group. Points within these reference ranges (e.g., controls, S6.1 and S6.2) indicate preserved morphology, while points outside (S1–S5) reflect marked alterations in melanocyte morphology and/or melanin content. (**D**) UMAP integrating all morphometric parameters reveals a clear separation of toxic fractions from controls and non-toxic fractions. Data points are color-coded by treatment; shapes indicate origin (squares = untreated control, circles = BGHO-1, cross = BGSJ2-8).

Our image analysis revealed that the tested fractions differed significantly not only in their impact on melanin content but also in their effects on melanocyte morphology (Figure 5A–C). Morphometric analysis demonstrated that treatments with S6.2 and S6.1 reduced pigmentation without altering solidity (Figure 5B) or area-to-perimeter ratio of melanocytes (Figure 5C) (*p* > 0.05, ANOVA, vs. control), confirming that the structural integrity of melanocytes was preserved upon the applied anti-melanogenic treatments. Importantly, melanocytes retained their characteristic star-like dendritic shape, closely resembling those of untreated embryos. These findings emphasize the biosafety and therapeutic potential of the tested postbiotics as non-toxic modulators of pigmentation. A similar effect was observed for the kojic acid treatment, in line with previous studies [54,56]. In contrast, treatments with hydroquinone or native overnight cultures (S1) disrupted melanocyte morphology, induced cell rounding, and led to a complete loss of dendricity, indicative of impaired melanocyte function. The deleterious effects of hydroquinone observed here are consistent with melanocytotoxic effects previously reported in mammalian models. Moreover, its topical use is associated with the irreversible loss of melanocytes. Safety concerns related to its mutagenic and hematologic toxicity have led to this remedy being banned in the EU, the USA, and Canada, and its use is strictly controlled in Australia [57].

To place these findings into a broader context, we next integrated melanocyte pigmentation and integrity parameters with toxicity readouts using clustering analysis and dimensionality reduction with UMAP (Figure 5D). This integrated analysis clearly delineated two well-defined categories of treatments. Group I comprised the safe and effective treatments—represented exclusively by fractions S6.1 and S6.2 from both strains, that produced a robust inhibition of melanogenesis while preserving melanocyte integrity and exhibiting no detectable adverse effects. In contrast, Group II encompassed melanocytotoxic treatments, including hydroquinone, kojic acid, and live probiotic cultures (S1–S3), all of which markedly reduced embryo pigmentation, but also were safe treatments with only minimal impact on melanin content or cellular morphology.

Taken together, these data demonstrate that specific heat-inactivated fractions of both probiotic strains, in particular S6.2, can effectively and safely reduce melanin synthesis. Our integrated zebrafish image-based analysis thus provides a high-resolution and phenotypically sensitive approach to distinguish effective and safe anti-melanogenic treatments from those associated with undesirable melanocytotoxic profiles, supporting the further development of selected fractions as promising postbiotic and biocompatible depigmenting agents.

### 2.3. Postbiotics of L. salivarius (BGHO-1) and L. paracasei (BGSJ2-8) Do Not Elicit Inflammatory Responses nor Neutropenia

To more comprehensively evaluate the potential use of the heat-inactivated postbiotic fractions S6.1 and S6.2 in skin depigmentation therapy, we further investigated their impact on the immune responses, focusing on inflammatory activation and the possible induction of neutropenia. For this purpose, we employed zebrafish embryos of the transgenic line *Tg*(*mpx*:GFP)i114 with fluorescently labeled neutrophils and monitored neutrophil numbers and fluorescence intensity after 48 h of treatment with fraction S6.1 (cytoplasmic content) or S6.2 (cell walls and membranes). As a positive control, lipopolysaccharide (LPS) from *Escherichia coli*, a potent inducer of inflammation widely used to study immune responses to Gram-negative bacteria and to screen for anti-inflammatory agents [58], was applied to parallel embryo groups.

**Figure 6:**
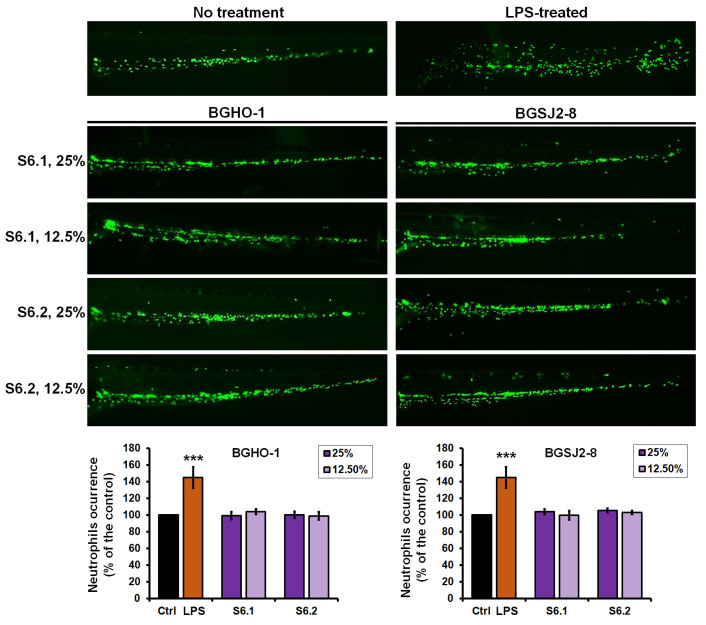
Immunotoxic responses in zebrafish embryos exposed to postbiotic fractions from *L. salivarius* (BGHO-1) and *L. paracasei* (BGSJ2-8). Fractions included cytoplasmic content (S6.1) and cell wall/membrane components (S6.2) of heat-inactivated bacterial cells were tested. Transgenic *Tg*(*mpx*:GFP)i114 embryos (*n* = 20 per treatment, three independent experiments) were treated with 12.5% and 25% doses of S6.1 and S6.2, and neutrophil counts in the caudal region were quantified relative to untreated controls. Lipopolysaccharide (LPS) from *E. coli* served as a positive inflammatory control. Statistically significant differences between control and treated groups are indicated (***, *p* < 0.001, ANOVA with Bonferroni test).

The results demonstrated that LPS treatment elicited a robust inflammatory response, with neutrophil numbers increasing by 45.2 ± 12.4% compared to controls (*p* < 0.01) (Figure 6). In contrast, treatment with the heat-inactivated fractions S6.1 and S6.2 from BGHO-1 and BGSJ2-8 neither altered neutrophil numbers (*p* = 0.781) nor affected fluorescence intensity (*p* = 0.911) relative to untreated embryos. These findings indicate that the tested fractions neither trigger inflammation nor induce immunosuppression (neutropenia), underscoring their favorable safety profile.

This immunological inertness is particularly relevant for their prospective use in dermatological and cosmetic formulations, where long-term topical application requires not only efficacy but also the absence of pro-inflammatory activity to prevent adverse reactions. Moreover, the lack of inflammatory activation is especially important in depigmentation therapy, since inflammation can additionally promote post-inflammatory hyperpigmentation and compromise therapeutic efficacy [59].

Data from this assay demonstrated that postbiotic fractions from *L. salivarius* BGHO-1 and *L. paracasei* BGSJ2-8 do not elicit an immunotoxic response at doses that effectively prevent pigmentation in zebrafish embryos, supporting their further evaluation as potential inhibitors of melanogenesis.

### 2.4. Novelty, Study Limitation, and Future Directions

Although previous studies have shown that certain probiotic strains can modulate melanogenesis (Table 1), most investigations examined only native cell-free supernatants and relied on simplified in vitro assays (mushroom tyrosinase or B16 cells), thereby limiting their full translational potential. Here, we identified *L. salivarius* BGHO-1 and *L. paracasei* BGSJ2-8 as new probiotic strains with notable potential to reduce melanin biosynthesis in vivo, while advancing understanding of the anti-melanogenic potential of a probiotic strain beyond single-fraction assessments.

Importantly, we implemented a multi-tier fractionation workflow that discriminates among native and heat-inactivated whole cultures, intact cells, secreted metabolites, and subcellular fractions (membrane versus cytoplasmic). This strategy enabled a comprehensive assessment of the true anti-melanogenic activity of each tested probiotic strain and identification of membrane-enriched postbiotic fractions as the most potent and biocompatible inhibitors of epidermal melanogenesis. Moreover, we combined a physiologically relevant, high-throughput zebrafish screening platform with imaging-based single-cell pigmentation analysis, allowing us to distinguish true anti-melanogenic effects from non-specific melanocytotoxicity, while simultaneously addressing systemic toxicity and immune responses—two aspects often overlooked in earlier work. This integrative approach can be applied to other probiotic strains to systematically explore their anti-melanogenic potential and accelerate identification of bioactive principles. Thus, our study complements prior reports and provides a tractable route for downstream molecular characterization of active constituents.

Nevertheless, this study has some limitations. Our results derive exclusively from the zebrafish model and should therefore be regarded as a foundational, preclinical step toward mechanistic and translational development of postbiotic-based modulators of melanogenesis rather than as finalized therapeutic products. To progress these findings, future work should include the following: (i) activity-guided fractionation and chemical characterization of active constituents (LC-MS/MS metabolomics, lipidomics and proteomics, and targeted analysis of membrane glycoconjugates, lipoteichoic acids and specific lipid species), (ii) validation of safety and efficacy in human-relevant model systems (primary human melanocytes, reconstructed epidermis, organoids and explant cultures) and, where appropriate, in rodent models as a preclinical standard, (iii) transcriptome and gene expression analysis to elucidate the molecular target(s), (iv) assessment of formulation and pharmacological properties relevant to topical use (stability, skin penetration, local tolerability, preservative compatibility and vehicle optimization, and (v) chronic-exposure and human-safety testing (irritation, sensitization and systemic exposure assessments) prior to consideration for cosmetic application. Integrating multi-omics compositional data with bioactivity readouts will be essential to identify active molecules and to create the appropriate product formulation.

## 3. Conclusions

This study demonstrates that heat-inactivated, membrane-enriched postbiotic fractions from *L. salivarius* BGHO-1 and *L. paracasei* BGSJ2-8 are promising, tractable bioactive candidates for modulating skin pigmentation. By combining multi-layer fractionation with in vivo zebrafish-based bioactivity profiling, single-cell imaging, and bulk melanin quantification, we established a comprehensive methodological framework to evaluate safety, efficacy, and mechanistic specificity. Using this integrative approach, we show that membrane-enriched postbiotic fractions produce dose-dependent inhibition of pigmentation while preserving melanocyte morphology and exhibiting a favorable in vivo safety profile compared to benchmark agents (hydroquinone, kojic acid). Moving beyond traditional small-molecule inhibitors, the postbiotic preparations offer a promising translational avenue for the development of safe, biologically inspired inhibitors of melanogenesis, paving the way for probiotic- and postbiotic-based advancements in dermatological research.

## 4. Materials and Methods

### 4.1. Literature Survey

In order to provide a comprehensive survey of probiotic bacterial strains with documented melanogenesis-inhibitory properties, we performed a systematic literature search that included studies reporting probiotic activity and examining the effects of live and inactivated probiotic cells, as well as their parabiotic and postbiotic derivatives. Importantly, we excluded studies investigating fermentation products enriched with probiotics, such as fermented milk, soy, or other food-based matrices, since our focus was restricted to preparations tested under controlled microbiological conditions.

### 4.2. Bacterial Strains and Culture Conditions

Two biochemically characterized probiotic strains, *Lactobacillus salivarius* BGHO-1 [45] and *Lactobacillus paracasei* BGSJ2-8 [46], were used in this study. Strains are part of the Laboratory collection of microorganisms at the Institute of Molecular Genetics and Genetic Engineering, University of Belgrade. The bacteria were cultivated in De Man-Rogosa-Sharpe (MRS) medium (Merck GmbH, Darmstadt, Germany) at 37 °C for 18 h.

The overnight cultures were used for obtaining a series of different fractions: the whole culture (S1), the cell-free supernatant (S2) obtained after centrifugation of the overnight culture at 13,000× *g* for 20 min (Eppendorf, 5315 D, Hamburg, Germany), and the bacterial pellet (S3) collected after separation of supernatant and washing three times in PBS. The corresponding heat-inactivated fractions were generated by incubating S1–S3 fractions at 95 °C for 30 min, giving S4 (heat-inactivated whole culture lysate), S5 (heat-inactivated culture supernatant), and S6 (heat-inactivated bacterial pellet—bacterial cell lysate).

To further dissect the composition of S6, the fraction was subjected to sonication on ice (10 s pulse + 30 s pause, three cycles), followed by centrifugation at 13,000× *g* for 30 min. This procedure separated the cytoplasmic fraction (S6.1, supernatant) from the cell wall/membrane fraction (S6.2, pellet). The obtained pellet (S6.2) was subsequently washed twice in PBS, centrifuged, and finally resuspended in the appropriate volume of embryo water.

### 4.3. In Vivo Toxicity Assessment in the Zebrafish Model

All experiments involving zebrafish (*Danio rerio*) embryos were performed in compliance with the European directive 2010/63/EU and the ethical guidelines for the care and use of laboratory animals of the Institute of Molecular Genetics and Genetic Engineering, University of Belgrade. Wild-type (AB) zebrafish embryos kindly provided by dr Ana Cvejic (Wellcome Trust Sanger Institute, Cambridge, UK) were raised to the adult stage in a temperature- and light-controlled zebrafish facility at 28 °C and a standard 14:10 h light-dark photoperiod. Fish were fed with commercial dry food (SDS200 and SDS300 granular food; Special Diet Services, Essex, UK, and TetraMin^TM^ flakes; Tetra, Melle, Germany) twice a day, and with *Artemia nauplii* daily.

The toxicity of probiotic-derived fractions (S1–S6.2) was evaluated in accordance with the general rules of the OECD Guidelines for the Testing of Chemicals 23 [60]. Embryos produced by pairwise mating were washed to remove debris, distributed at 10 per well into 24-well plates containing 1 mL of E3 medium (5 mM NaCl, 0.17 mM KCl, 0.33 mM CaCl_2_, and 0.33 mM MgSO_4_ in distilled water), and maintained at 28 °C. For assessing acute (lethal), inner organs and developmental (teratogenic) toxicity, the embryos were treated with the five different concentrations of the tested fractions (50%, 25%, 12.5%, 6.25% and 3.13%) at 6 h post fertilization (hpf) stage, ensuring thus high sensitivity to applied treatments. Treated embryos were inspected every day under a stereomicroscope (Carl Zeiss™ Stemi 508 doc Stereomicroscope, Jena, Germany) for the appearance of the 18 toxicity-relevant endpoints until 120 hpf (Table S1). Specifically, the effect on pigmentation (melanogenesis) was inspected at 48 hpf. Dead embryos were recorded and discarded every 24 h. E3 medium was used as the negative control. The experiment was performed three times using 20 embryos per concentration (*n* = 60 in total). At 120 hpf, embryos were anesthetized by the addition of 0.1% (*w*/*v*) tricaine solution (Sigma-Aldrich, St. Louis, MO, USA), photographed, and inactivated by freezing at −20 °C for ≥24 h.

### 4.4. In Vivo Assessment of Anti-Melanogenic Activity in the Zebrafish Model

The ability of probiotic cells and derived fractions to inhibit melanogenesis was evaluated in vivo using zebrafish embryos (AB strain). Embryos at the 6 hpf stage were exposed to four different concentrations (6.25%, 12.5%, 25%, and 50%) of the tested compounds for 48 h. Experiments were carried out in 24-well plates, with 10 embryos per well, and incubated at 28 °C. A total of 50 embryos per concentration were used. The experiment was performed three times (*n* = 150 in total). Following treatment, embryos were examined under a light microscope (Motic SMZ140 Series, Xiamen, China) to assess skin pigmentation and the morphological characteristics of melanocytes. Kojic acid (100 and 2000 µg/mL) and hydroquinone (12.5 µg/mL) served as positive controls. For quantitative melanin analysis, pooled samples of 50 embryos per concentration were subjected to sonication on ice (30 s of sonication followed by 30 s of cooling, repeated for three cycles) to release melanin from melanocytes and melanosomes. The samples were then centrifuged (10 min at 12,000× *g*), the supernatant was discarded, and the pellet was resuspended and heated in 1 mL of 1 M KOH at 95 °C for 30 min to dissolve the released melanin. After a final centrifugation, the absorbance of the melanin-containing supernatant was measured at 490 nm using a BioTek Epoch Microplate Spectrophotometer (Winooski, VT, USA). Melanin content was expressed relative to the control group, with pigmentation in untreated embryos defined as 100%.

### 4.5. Immunotoxicity Assessment In Vivo Using Transgenic Zebrafish

To investigate the possible in vivo immunotoxic effects (inflammation and neutropenia) of anti-melanogenic fractions of fractions S6.1 and S6.2, embryos of the transgenic zebrafish line *Tg*(*mpx:*GFP)i114 [61], whose neutrophils express green fluorescent protein (GFP), were used. Experiments were conducted in 24-well microtiter plates, with 10 embryos per concentration in each well. The experiment was performed three times (*n* = 30 in total). At 72 hpf, when neutrophils were already formed, embryos were treated with the given fractions at effective and safe melanogenesis-inhibitory concentrations (12.5% and 25%). After 48 h of incubation at 28 °C, embryos were anesthetized with tricaine (200 µg/mL) and observed under a fluorescence microscope. Neutrophil occurrence was analyzed according to fluorescence intensity in the caudal region using the ImageJ program. Fluorescence levels in the treated groups were compared to those in the untreated control embryos, where the neutrophil signal was considered 100%. As a positive control for inflammation, lipopolysaccharide (LPS; Sigma-Aldrich, St. Louis, MO, USA) from *Escherichia coli* serotype O111 was used at a concentration of 50 µg/mL, given its established ability to induce a robust inflammatory response in zebrafish embryos.

### 4.6. Image-Based Analysis of Melanin Content and Melanocyte Morphology

To assess the anti-melanogenic efficacy of probiotic-derived treatments, images of zebrafish embryos were analyzed using the ImageJ program v1.54a [62]. Regions of interest and individual melanocytes were manually selected in ImageJ, followed by automated thresholding and quantitative analysis of pigmentation and morphology. All segmentation outputs were visually inspected to confirm accuracy before data extraction. The pigmented area within the yolk region—defined as the region of interest (ROI)—was quantified by converting RGB images to 8-bit grayscale and delineating the ROI using the oval selection tool. The mean grayscale intensity within the ROI was measured, and melanocytes were isolated by thresholding based on intensity contrast. The reduction in pigmentation was calculated as the difference in mean intensity between the total ROI and the pigmented areas, normalized to the untreated control group, and expressed as a percentage reduction.

To examine the impact of treatments on melanocyte structure, two morphometric parameters were extracted from segmented melanocyte images: solidity (the cell area relative to the convex hull area) and the area-to-perimeter ratio. These descriptors provide quantitative insight into cellular compactness and dendricity—key features of melanocyte integrity.

For each treatment condition, six embryos were analyzed, and three to five melanocytes per embryo were evaluated for melanin content and morphological parameters. Melanin content data from individual embryos across treatment types were expressed as a percentage of control values and visualized using violin plots to illustrate variability and distribution across groups. To integrate pigmentation and morphological descriptors, we applied Uniform Manifold Approximation and Projection (UMAP) using the umap-learn Python package (v0.5.3) with parameters: n_neighbors = 10, min_dist = 0.1, n_components = 2, and random_state = 42. This dimensionality reduction technique allowed the unsupervised clustering of treatments based on their combined phenotypic profiles, enabling clear separation of effective and safe treatments from those that were effective but associated with cytotoxic effects.

### 4.7. Statistical Data Analysis

Statistical analyses were performed in IBM SPSS Statistics v21. Data are presented as mean ± standard deviation (SD). After normality of data distribution (Shapiro–Wilk test) and homogeneity of variance (Levene’s test) were assessed. The group comparisons (treated vs. untreated) were performed using one-way ANOVA followed by Bonferroni post hoc test; two-sided *p* < 0.05 was considered statistically significant. For pooled melanin-extraction assays, three independent biological experiments were performed with a total of 150 embryos. For the neutrophil-occurrence assay, three independent experiments were performed with a total of 30 embryos.

## Figures and Tables

**Table 1:** The summary of probiotic strains and their derived fractions with anti-melanogenic activity.

Probiotic Strain	Active Fraction(s)	Cultivation Conditions	Melanogenesis Testing Model	Active Substance(s)	Toxicity Model	Ref.
*Bifidobacterium adolescentis* BCRC 14658	Cell-free culture supernatant	MRS + cysteine, anaerobic (10% CO_2_, 10% H_2_, 80% N_2_)	Mushroom tyrosinase B16F10 tyrosinase	/	B16F10 cells	[36]
*Bifidobacterium bifidum* IDCC 4201	Culture supernatant	MRS, anaerobic	Mushroom tyrosinase α-MSH-stimulated B16F10 cells	Multiple metabolites, including phenyllactic acid	B16F10 cells	[30]
*Bifidobacterium longum* subsp. *iuvenis* YSG	Fermentation metabolites	anaerobic fermentor with MRS broth	Mushroom tyrosinase UVB-induced and α-MSH-stimulated B16F10 cells	5-hydroxyindole-2-carboxylic acid, aconitic acid glycolic acid	NIH-3T3 cells HaCaT cells	[39]
*Bifidobacterium longum* ZJ1	Whole cell lysate Bacterial pellet Cell-free supernatant	MRS + cysteine, anaerobic	Mushroom tyrosinase α-MSH-stimulated B16F10 cells Zebrafish embryos	n.d.	B16F10 cells HaCaT cells	[29]
*Lactobacillus plantarum* IDCC 3501 (*Lactiplantibacillus plantarum*)	Cell-free culture supernatant	MRS, anaerobic	Mushroom tyrosinase α-MSH-stimulated B16F10 cells	Multiple metabolites, including phenyllactic acid	B16F10 cells	[30]
*Lactobacillus plantarum* (KCCM)	Lipoteichoic acid	MRS	Mushroom tyrosinase α-MSH-stimulated B16F10 cells	Lipoteichoic acid	B16F10 cells	[37]
*Lactobacillus plantarum* TWK10	Lyophilized ethanol extract	Black soybeans	UVB-exposed nude mice	n.d.	n.d.	[32]
*Lactobacillus plantarum* WB326	Lyophilized cell-free culture supernatant	MRS, anaerobic	Mushroom tyrosinase α-MSH-stimulated B16F10 cells	Organic acids	B16F10 cells	[28]
*Lactobacillus acidophilus* KCCM12625P	Heat-inactivated overnight culture	Not specified	Mushroom tyrosinase α-MSH-stimulated B16F10 cells	n.d.	B16F10 cells HaCaT cells	[35]
*Lactobacillus gasseri* BNR17	Cell-free culture supernatant	MRS, aerobic	α-MSH-stimulated B16F10 cells	n.d.	B16F10 cells HaCaT	[31]
*Lactobacillus kunkeei* NCHBL-003 (*Apilactobacillus kunkeei*)	Cell-free culture supernatant	DMEM, aerobic	α-MSH-stimulated B16F10 cells	n.d.	B16F10 cells	[27]
*Lactobacillus rhamnosus* ATCC 7469 (*Lacticaseibacillus rhamnosus*)	Heat-inactivated whole cell culture	Cell growth media, 5% CO_2_	UVB-irradiated human epidermal melanocytes—HEM	n.d.	HEM	[38]
*Lactobacillus brevis* WB2810 (*Levilactobacillus brevis*)	Cell-free culture supernatant	MRS	α-MSH-stimulated B16F10 cells	Organic acids	B16F10 cells	[28]
*Lactobacillus fermentum* JNU532 (*Limosilactobacillus fermentum*)	Cell-free culture supernatant	MRS	Mushroom tyrosinase B16F10 cells	n.d.	B16F10 cells	[33]
*Pediococcus acidilactici* PMC48	Cell-free culture supernatant	MRS, anaerobic/aerobic	Mushroom tyrosinase α-MSH-stimulated B16F10 cells	n.d.	B16F10 cells	[34]

## Data Availability

The original contributions presented in this study are included in the article/Supplementary Materials. Further inquiries can be directed to the corresponding author.

## Supplementary Materials

The following supporting information can be downloaded at: https://www.mdpi.com/article/10.3390/molecules30204134/s1. Table S1: Lethal and teratogenic effects observed in zebrafish (*Danio rerio*) embryos at different hours post fertilization (hpf). Figure S1: The dose-dependent inhibition of melanogenesis in zebrafish embryos exposed to the different fractions (S1–S6.2) derived from overnight cultures of the probiotic strains *Lactobacillus salivarius* BGHO-1; Figure S2: The dose-dependent inhibition of melanogenesis in zebrafish embryos exposed to the different fractions (S1–S6.2) derived from overnight cultures of the probiotic strains *Lactobacillus paracasei* BGSJ2-8.
