# Supplementary material for: Targeting Melanogenesis with Postbiotics: An Integrated Zebrafish-Based Assessment of Lactobacillus salivarius BGHO-1 and Lactobacillus paracasei BGSJ2-8
Source: Molecules. 2025 Oct 20;30(20):4134. doi: 10.3390/molecules30204134 (PMC12567018; doi:10.3390/molecules30204134)
Supplement: Supplementary file 1 [file molecules-30-04134-s001.zip › molecules-3915769-supplementary.pdf]

Targeting Melanogenesis with Postbiotics: an integrated Zebrafish-Based Assessment of *Lactobacillus salivarius* BGHO-1 and *Lactobacillus paracasei* BGSJ2-8

Gabor Katona[1], Natasa Jovanovic Ljeskovic[1], Ivana Strahinic[2], Nemanja Stanisavljevic[2], Sladjana Vojvodic[1], Jelena Djuris[3], Aleksandar Pavic[2*]

[1] Faculty of Pharmacy Novi Sad, University Business Academy in Novi Sad, Heroja Pinkija 4, the Republic of Serbia

[2] Institute of Molecule Genetics and Genetic Engineering, University of Belgrade, Vojvode Stepe 444a, 11042 Belgrade, the Republic of Serbia

[3] University of Belgrade - Faculty of Pharmacy, Vojvode Stepe 450, 11221 Belgrade, the Republic of Serbia

Content:

Table S1. Lethal and teratogenic effects observed in zebrafish (*Danio rerio*) embryos at different hours post fertilization (hpf).

Figure S1. The dose-dependent inhibition of melanogenesis in zebrafish embryos exposed to the different fractions (S1-S6.2) derived from overnight cultures of the probiotic strains *Lactobacillus salivarius* BGHO-1

Figure S2. The dose-dependent inhibition of melanogenesis in zebrafish embryos exposed to the different fractions (S1-S6.2) derived from overnight cultures of the probiotic strains *Lactobacillus paracasei* BGSJ2-8.

Table S1. Lethal and teratogenic effects observed in zebrafish (*Danio rerio*) embryos at different hours post fertilization (hpf).

Category	Toxicological parameters	Exposure time (hpf)				
		24	48	72	96	120
**Lethal effect**	Coagulated eggs[a]	●	●	●	●	●
	Lack of the heart beating	●	●	●	●	●
	Non-detachment of the tail	●	●	●	●	●
	Lack of somite formation	●	●	●	●	●
**Teratogenic effect**	Malformation of head	●	●	●	●	●
	Malformation of eyes[b]	●	●	●	●	●
	Malformation of sacculi/otoliths[c]	●	●	●	●	●
	Malformation of chorda[d]	●	●	●	●	●
	Malformation of tail[e]	●	●	●	●	●
	Scoliosis/lordosis	●	●	●	●	●
	Yolk edema[f]	●	●	●	●	●
	Growth retardation[g]		●	●	●	●
	Hatching[h]			●	●	●
	Swimmbladder development[i]					●
**Hepatotoxicity**	Yolk absorption[j]			●	●	●
	Liver darkening[k]			●	●	●
**Cardiotoxicity**	Pericardial edema[l]		●	●	●	●
	Heart beating rate (beat/min)[m]					●

[a] No clear organs structure is recognized
[b] Malformation of eyes was recorded for the retardation in eye development and abnormality in shape and size.
[c] Presence of none, one or more than two otoliths per sacculus, as well as reduction and enlargement of otic vesicles
[d] The abnormality in notochord shape
[e] Tail was bent, twisted or shorter than to control embryos as assessed by optical comparison
[f] Enlargement of the yolk sac
[gf] Growth retardation was recorded by comparing with the control embryos in a body length (after hatching)
[h] Embryos hatching in a period from 72 hpf to 120 hpf stage
[i] The presence, reduced size or absence of swimmbladder
[j] The resorption of yolk
[k] The change in liver color and dark color appearance
[l] An appearance of pericardial sac enlargment
[m] The number of beats within 30 sec

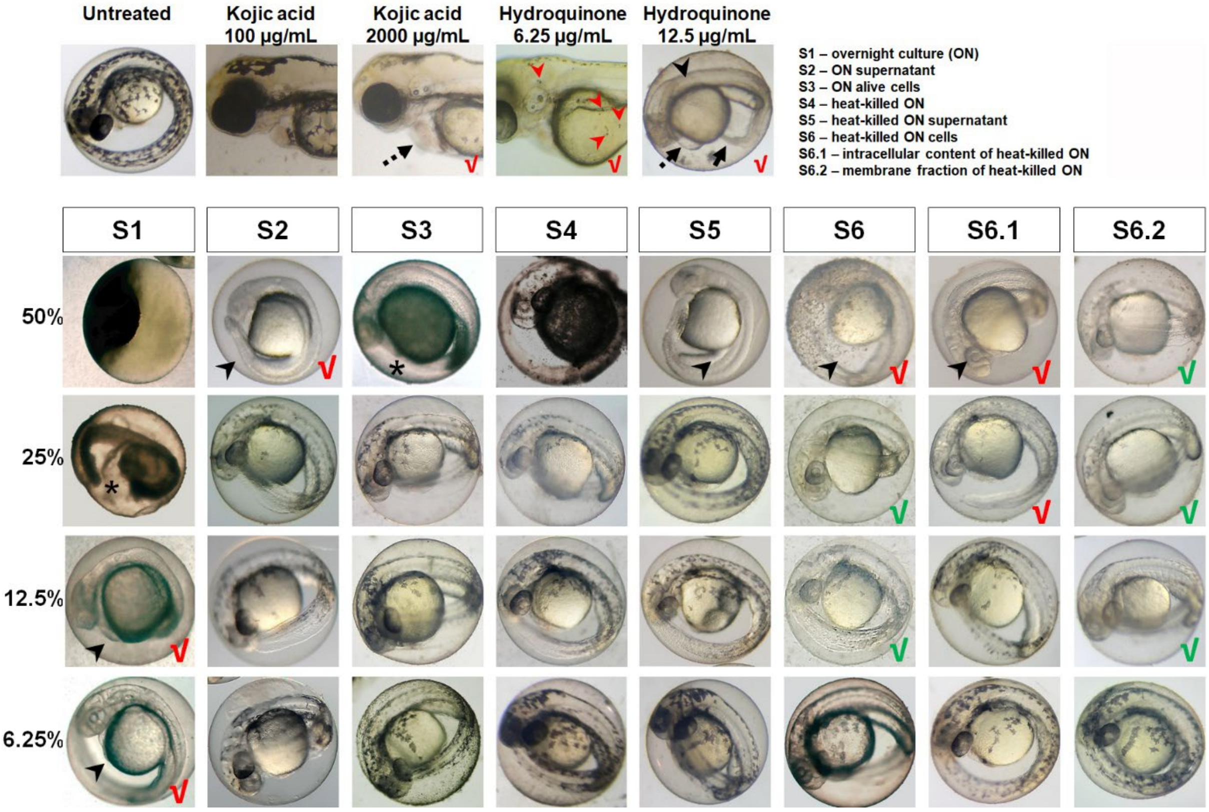

Supplementary Figure S1. The dose-dependent inhibition of melanogenesis in zebrafish embryos exposed to the different fractions (S1-S6.2) derived from overnight cultures of the probiotic strains *Lactobacillus salivarius* BGHO-1. Non-toxic fractions that reduced embryo pigmentation without adverse effects are marked in green. Toxic fractions are marked in red and provoked multiple toxic effects, including embryo mortality (asterisk), impaired growth and development (arrowhead), tail necrosis (solid arrow), pericardial edema (dashed arrow), and melanocytotoxicity (red arrow), are indicated on the corresponding images.

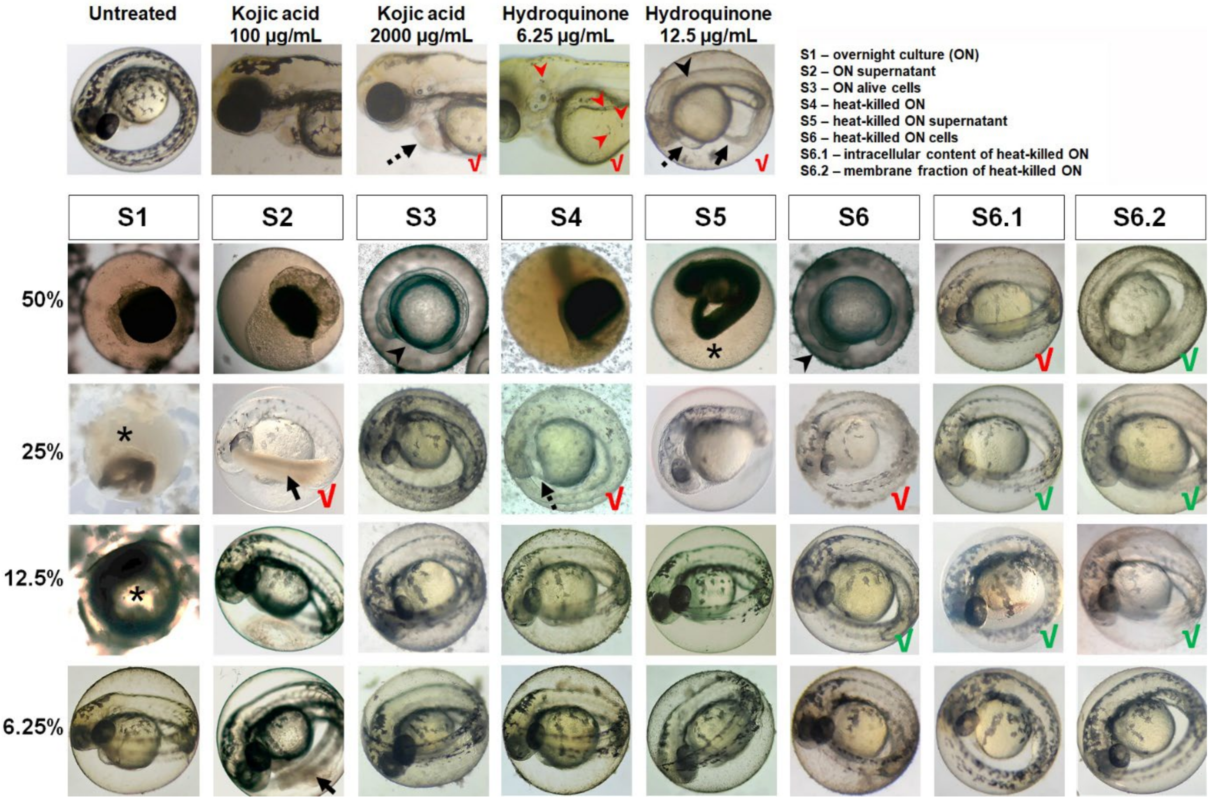

Supplementary Figure S2. The dose-dependent inhibition of melanogenesis in zebrafish embryos exposed to the different fractions (S1-S6.2) derived from overnight cultures of the probiotic strains *Lactobacillus paracasei* BGSJ2-8. Non-toxic fractions that reduced embryo pigmentation without adverse effects are marked in green. Toxic fractions are marked in red and provoked multiple toxic effects, including embryo mortality (asterisk), impaired growth and development (arrowhead), tail necrosis (solid arrow), pericardial edema (dashed arrow), and melanocytotoxicity (red arrow), are indicated on the corresponding images.
